# Bioavailability of a novel anti-influenza agent, onradivir granules, in healthy Chinese adults: a first-in-human study

**DOI:** 10.3389/fphar.2026.1905152

**Published:** 2026-09-01

**Authors:** Xiaoyi Yi, Yuting Liu, Xiaosong Wang, Yahui Peng, Jian Fu, Youyun Li, Haijun Li, Hong Zhang

**Affiliations:** 1 Department of Phase I Clinical Trial Unit, Jiangxi Cancer Hospital & Institute (The Second Affiliated Hospital of Nanchang Medical College), Nanchang, China; 2 Guangdong Raynovent Biotech Co., Ltd., Guangdong, China; 3 Jiangxi Key Laboratory of Translational Research for Cancer, Jiangxi Clinical Research Center for Cancer, Nanchang, China

**Keywords:** first-in-human bioavailability, highly variable drug, onradivir granules, pediatric formulation, pharmacokinetic parameters, relative bioavailability

## Abstract

**Background:**

Onradivir (ZSP1273) is a novel inhibitor of the RNA polymerase PB2 subunit of influenza A virus, developed for the treatment of acute uncomplicated influenza A. The onradivir tablet, launched in 2023, is intended for adult patients and is not suitable for children. This project aims to evaluate the pharmacokinetic profile of the onradivir granule formulation, providing evidence to support its use in the target pediatric population as well as in adult patients.

**Methods:**

This study adopted a open, four-period, repeated crossover design. Thirty-two healthy adult males received 200 mg doses of either the onradivir granules or onradivir tablets under fasting conditions. To quantify the plasma concentration of onradivir using an LC-MS/MS method and evaluate its pharmacokinetic parameters.

**Results:**

During the trial, no SAEs or SUSARs occurred in any participants. The coefficient of variation for C_max_ of the onradivir tablets was 74.91%, indicating high variability and necessitating the use of the RSABE method for evaluating C_max_ equivalence between the two formulations. Within-subject standard deviation of the reference formulation (S_WR_) value of C_max_ was 0.667, exceeding the cutoff of 0.294. C_max_ met both acceptance criteria: its 95% upper confidence bound, at −0.1769, was <0, and the point estimate of the least-squares geometric mean ratio is 81.58%, fell within the 80.00%–125.00% range, meeting the RSABE equivalence determination criteria; The coefficients of variation for AUC_0-t_ and ${\text{AUC}}_{0-\infty }$ of the onradivir tablets were 18.11% and 15.47%, respectively; therefore, the ABE method was employed to assess the equivalence of AUC_0-t_ and ${\text{AUC}}_{0-\infty }$ . The geometric mean ratios and 90% confidence intervals for AUC_0-t_ and ${\text{AUC}}_{0-\infty }$ were 98.13% and 97.52%, with their corresponding 90% confidence intervals of 93.91%–102.54% and 93.22%–102.03%, respectively, all falling within the 80.00%–125.00% range, meeting the ABE equivalence determination criteria.

**Conclusion:**

The two formulations demonstrated comparable bioavailability and met the criteria for bioequivalence.

**Clinical Trial Registration:**

https://www.chinadrugtrials.org.cn/clinicaltrials.searchlistdetail.dhtml, identifier CTR20242821.

## 1 Introduction

Influenza A is one of the most common and highly contagious infectious diseases in humans. According to estimates from the World Health Organization (WHO), there are approximately 1 billion cases of seasonal influenza globally each year, including 3–5 million severe cases, resulting in 290,000 to 650,000 respiratory deaths annually (World Health Organization, 2025). Influenza A viruses possess 18 subtypes of the surface glycoprotein hemagglutinin (HA) (H1–H18) and 11 subtypes of neuraminidase (NA) (N1–N11). These viral proteins drive influenza pandemics and represent a major global public health challenge (Harrington et al., 2021). Some patients with influenza A may develop severe influenza due to complications such as pneumonia or exacerbation of underlying diseases. They may die from complications such as acute respiratory distress syndrome (ARDS), acute necrotizing encephalopathy, or multiple organ dysfunction (Centers for Disease Control and Prevention, 2023b; Nguyen et al., 2024; Valenzuela-Sanchez et al., 2024; Bartolini et al., 2024; Shah et al., 2022). Due to the high mutation rate of influenza viruses and the occurrence of viral recombination, existing antiviral drugs struggle to cope with the constantly evolving influenza strains, leading to viral escape from vaccine effects (World Health Organization, 2024). All children under the age of 5 are considered high-risk groups for severe influenza (Nayak et al., 2021; Hu et al., 2023). One study has shown that there are 9,243–105,690 influenza-related respiratory deaths in children under 5 years old each year (Iuliano et al., 2018; Ruf and Knuf, 2014).Given the triple vulnerability of children (especially infants under 5 years old) during influenza epidemics: high infection rate (annual incidence of 20%–30%) (Fraaij and Heikkinen, 2011), high severe case conversion rate (Nair et al., 2011), and high household transmission risk (secondary attack rate of 38%) (Tsang et al., 2016). The WHO has explicitly classified children under 5 as a high-risk group for severe influenza. This underscores the clinical necessity and public health urgency of developing child-friendly anti-influenza A medications. Therefore, the development of novel anti-influenza drugs with innovative mechanisms of action and pediatric-appropriate formulations is critically urgent to address the growing unmet public health and clinical needs.

The currently clinically used anti-influenza drugs mainly include neuraminidase inhibitors (oseltamivir) and RNA polymerase inhibitors (baloxavir mesylate), etc. However, the issue of resistance is becoming increasingly prominent. The M2 ion channel inhibitors (amantadine and rimantadine) are no longer recommended for the treatment of influenza A due to widespread resistance (Kumar and Sakharam, 2024). Oseltamivir, as the most widely used anti-influenza drug in the past two decades, also requires attention to its resistance issue (Takashita et al., 2024). Additionally, the new-generation RNA polymerase inhibitor baloxavir mesylate also faces the problem of low resistance barriers (Takashita et al., 2025). Therefore, it is urgent to develop antiviral drugs with novel mechanisms of action and no cross-resistance to existing drugs. The PB2 subunit of the influenza virus RNA polymerase is highly conserved among different influenza viruses and is an important target for anti-influenza drugs.

Onradivir tablet is a innovative drugs, which is a novel RNA polymerase PB2 inhibitor targeting influenza A virus. It inhibits the normal initiation of the replication function of the RNA polymerase complex by binding to the PB2 subunit, thereby inhibiting various functions such as the transcription and replication of the viral genome in the viral life period, achieving an antiviral effect against influenza A (Takashita, 2021). The onradivir tablet was approved for marketing by the National Medical Products Administration (NMPA) of China in December 2023, with the indication for the treatment of adult patients with uncomplicated influenza A. However, the onradivir tablet formulation is not well-suited to meet the needs of a high-risk population for severe influenza—the pediatric population. Consequently, Guangdong Raynovent Biotech Co., Ltd. has developed a new formulation, onradivir granules, designed for ease of use in pediatric patients. This granule formulation not only addresses the clinical requirement for dose adjustments across different pediatric age groups (National Department of Health, 2024). But also provides a better dosing option for adult patients who have difficulty swallowing tablets.

In this study, the primary pharmacokinetic parameters of the granule and tablet formulations were compared to evaluate the relative bioavailability of the granules. By analyzing the differences in these key parameters between the two formulations, the findings provide data to support the registration and marketing of the granule formulation.

## 2 Materials and methods

### 2.1 Ethical review

This clinical study was approved by the Medical Ethics Committee of Jiangxi Cancer Hospital (Approval Number: 2024090-YW090; Date of Approval: 19 July 2024), and written informed consent was obtained from all participants. The study was conducted in accordance with the ethical standards for human research set forth in the Declaration of Helsinki and its amendments, the International Conference on Harmonisation (ICH) Good Clinical Practice guidelines, and the relevant clinical trial guidelines recommended by the National Medical Products Administration (NMPA) of China. This trial has been registered on the Drug Clinical Trial Registration and Information Disclosure Platform (Registration Number: CTR20242821, Registered on 02 August 2024, https://www.chinadrugtrials.org.cn/clinicaltrials.searchlistdetail.dhtml). The study reported following the Consolidated Standards of Reporting Trials (CONSORT) extension for Bioequivalence Studies (Hopewell et al., 2025). The completed checklist is provided as Supplementary Material.

### 2.2 Study drug

Test formulation (T): Onradivir Granules: 200 mg, Batch No.: BV24002, manufactured by Bostal Drug Delivery Co., Ltd. Preparation and administration process of ZSP1273 granule medication: (1) Add the medication to the container, then measure 20 mL of warm water with a measuring cylinder and pour it into the container. Stir for about 45 s and then take it orally. (2) The residues (the stirring rod and the cup wall) need to be rinsed. Take about 10 mL of warm water each time for rinsing until there is no obvious residue in the container. After each rinsing, let the subject take it immediately, and each rinsing should be completed within about 30 s (3) Finally, the subject should take the remaining warm water again. The total amount of warm water taken during the single granule medication process is 240 mL. The entire medication process should be completed within 1.5 min.

Reference formulation (R): Onradivir Tablets: 200 mg, Batch No.: 230802, manufactured by Guangdong Huanan Pharmaceutical Group Co., Ltd. ZSP1273 tablet administration process: 240 mL warm water, to be taken all at once.

Both T and R were provided by Guangdong Raynovent Biotech Co., Ltd.

### 2.3 Study design

This is a randomized, open-label, single-dose, four-period, two-sequence, repeated crossover, and fasting-administered clinical study.

Based on previous studies, the individual intra-subject coefficient of variation of C_max_ for ZSP1273 granules in the fasting state is 42.75%, which is a highly variable drug. This study adopts the FDA-recommended RSABE design to ensure that the Type I error rate is within an acceptable level. Assuming a one-sided α = 0.05 and β = 0.1, with a preset intra-subject coefficient of variation of 40%, and a ratio of the mean values of the test formulation and the reference formulation of 0.90, the sample size was calculated to be 24 cases using SAS (version 9.4). Considering a minimum 20% dropout rate among the subjects and the requirement of the regulatory authorities to complete at least 24 cases, this trial plans to include 32 subjects.

All trial participants were required to complete physical examinations, vital signs (body temperature, pulse, and seated blood pressure), 12-lead electrocardiogram, and specified laboratory tests (blood routine, urine analysis, blood biochemistry, coagulation function tests, infectious disease tests, etc.) before enrollment. Thirty-two eligible healthy Chinese males were selected.

**FIGURE 1:**
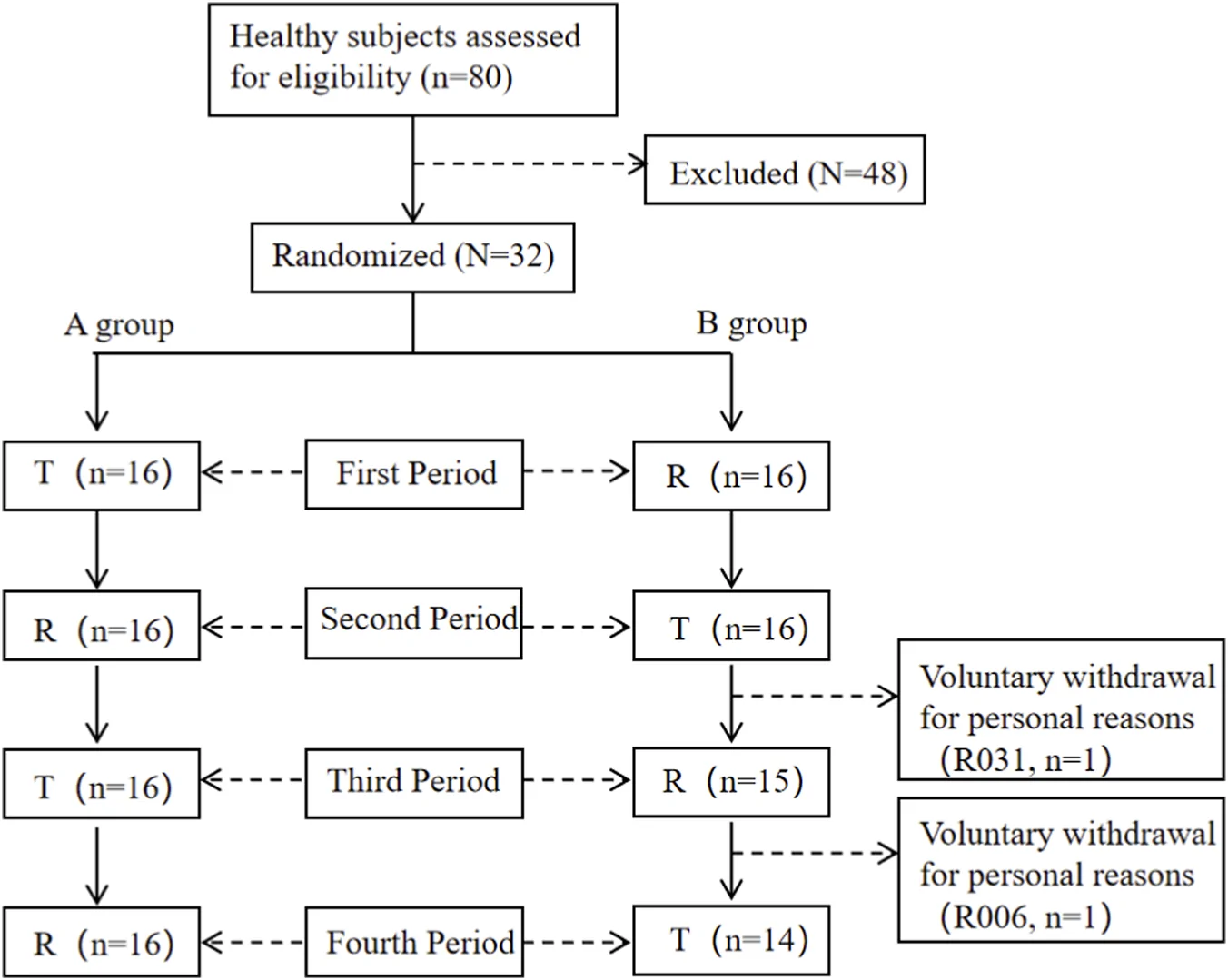
The study flow chart.

This trial uses the block randomization method to generate a random allocation sequence. The statistical unit uses the SAS software (version 9.4) to create a random table and divides the 32 screened participants into group A (T-R-T-R group) and group B (R-T-R-T group) in a 1:1 ratio. According to the screening numbers obtained by the participants, the random numbers are assigned in ascending order from smallest to largest. The random numbers for the participants consist of three digits (R001–R032). The randomization information is kept blinded during the analysis of biological samples and the transmission of related data, while the other investigators are not blinded. Each group consisted of 16 participants. After a 7-day washout period, the two groups were crossed over for administration (Figure 1). During the trial, the unblinded pharmacist provided coded medications to the research nurse according to the study phase. The research nurse subsequently administered these medications to the participants. Eligible subjects were healthy Chinese male volunteers aged 18–50 years, with a body weight of ≥50 kg and a body mass index (BMI) of 19.0–26.0 kg/m^2^. The time when the first trial participant signed the informed consent form was 2 August 2024, and the last trial participant completed the visit on 12 September 2024. The participants in the trial collected venous blood (3 mL per tube, with K2-EDTA as the anticoagulant in the collection tubes) before each administration cycle (0 h) and at 15 min, 30 min, 1 h, 1.5 h, 2 h, 3 h, 4 h, 6 h, 8 h, 12 h, 24 h (D2), 36 h (D2), 48 h (D3), and 72 h (D4) after administration. After collection, the blood samples were centrifuged at 1700 g for 10 min at 4 °C, and the separated plasma samples were stored at −80 °C for testing. The entire research process was carried out in the Department of Phase I Clinical Trial Unit of Jiangxi Cancer Hospital. The participants completed all the procedures under the medical security provided by the doctors.

### 2.4 Standardized management of onradivir granule administration and taste evaluation of onradivir granules

To ensure the standardization of the administration of the granules to the subjects, Department of Phase I Clinical Trial Unit has established a “Standard Operating Procedure for the Administration of onradivir Granules in Clinical Trials.” This SOP standardizes and provides standardized training on aspects such as the dissolution of the granules, stirring steps, and the method of administration. By doing so, it minimizes the potential differences in results caused by variations in the experimental procedures, thereby ultimately ensuring the accuracy of the evaluation of formulation differences. Furthermore, since the main target population of this formulation includes children, in order to ensure the compliance of the pediatric population in taking the medication, during the trial period, the VAS scoring scale was used to evaluate the taste of the granules. At present, there is no universally recognized “gold standard” scale for evaluating the taste of pediatric medications. Therefore, the research team of this project developed a self-made visual analogue scale (VAS) to conduct exploratory evaluations of the taste after taking the test formulation and before rinsing the mouth. The composition of the self-made VAS questionnaire used in this study - consisting of palatability evaluation (5-level classification VAS) and grittiness (100-mm continuous VAS), at the same time, record the behavioral responses (refusal to take medication, vomiting, retching, nausea, etc.). The higher the score for palatability evaluation, the better the subjective acceptance; the higher the score for grittiness, the stronger the perceived particle sensation. Detailed scoring operation instructions, scale items, and filling guidelines have been fully presented in Supplementary Material 1.

### 2.5 Safety assessment

The safety assessment of this project includes vital sign monitoring (body temperature, pulse, sitting blood pressure), 12-lead electrocardiogram, blood routine test, urine analysis, blood biochemistry, coagulation function test, and four items of infectious disease screening. These are all laboratory tests.

### 2.6 Method validation

In this study, ZSP1273-D4 (onradivir-D4) was used as the internal standard (IS), and an LC-MS/MS method was employed for the determination of onradivir plasma concentrations. During the full method validation, quality control (QC) samples were prepared at five concentration levels: lower limit of quantification QC (LLOQ QC) at 4.00 ng/mL, low QC (LQC) at 12.0 ng/mL, medium QC (MQC1) at 300 ng/mL, medium QC (MQC2) at 5,000 ng/mL, and high QC (HQC) at 7,500 ng/mL. Six replicates at each concentration level were analyzed to evaluate Intra-batch and Inter-batch precision and accuracy. Matrix effect and stability were assessed using LQC and HQC samples. Recovery was evaluated using LQC, MQC1, and HQC samples.

### 2.7 Blood drug concentration determination

The concentration range of onradivir in plasma is 4.00 - 10,000 ng/mL. The quantitative results use the peak area ratio of onradivir and IS as the vertical coordinate, and onradivir concentration as the horizontal coordinate. After performing linear regression with a weight factor of 1/x2, the quantitative determination of onradivir drug concentration in the plasma of the test participants after medication is conducted. The chromatograms of the analyte and the internal standard, as well as the chromatographic peak integration, are processed by the software Analyst 1.6.3 (AB Sciex). After optimizing the integration parameters, the target chromatographic peaks are automatically integrated, and individual chromatographic peaks cannot be integrated separately or manually.

### 2.8 Pharmacokinetic analysis

The pharmacokinetic parameters of this study were calculated using the Phoenix WinNonlin V8.4 software (Pharsight Corp, Mountain View, United States), employing a non-compartmental model (NCA) to determine the pharmacokinetic parameters of onradivir in plasma, including the main PK parameters (C_max_, AUC_0-t_, ${\text{AUC}}_{0-\infty }$ ) and the secondary PK parameters (T_max_, t_1/2_, AUC__%Extrap_, Vd/F, CL/F, F); the results were statistically analyzed using the SAS V9.4 software (SAS Institute, Cary, United States), including the full analysis set (FAS), the pharmacokinetic concentration set (PKCS), the pharmacokinetic parameter set (PKPS), the bioavailability set (BAS), and the safety set (SS). In this study, two subjects prematurely withdrew at the third and fourth cycles respectively. However, all of them had completed the full PK sampling for the previous cycles before withdrawing. Therefore, there were still evaluable pharmacokinetic data available. The missing pharmacokinetic concentration data were not interpolated in any way. This approach complies with the guidelines of the FDA, EMA, and NMPA regarding the bioequivalence studies, which do not recommend interpolating missing PK data.

### 2.9 Bioavailability assessment

Bioequivalence was assessed using the two one-sided t-tests (TOST) with a one-sided α of 0.05, and all other statistical tests were performed using a two-sided α of 0.05. BAS included at least one dosing cycle and had at least one evaluable pharmacokinetic parameter (AUC_0-t_). This dataset was used to infer the relative bioavailability and pharmacokinetic characteristics of the test formulation compared to the reference formulation. The C_max_, AUC_0-t_, and ${\text{AUC}}_{0-\infty }$ of T and R were transformed by natural logarithm and analyzed using a mixed-effects model. The variance analysis model included the dosing sequence, formulation factor, and dosing cycle as fixed effects, while the subjects and formulation factor were considered as random effects.

When the within-subject standard deviation of a pharmacokinetic parameter (C_max_, AUC_0-t_, or ${\text{AUC}}_{0-\infty }$ ) for the reference formulation was less than 0.294, ABE was used for evaluation. If the 90% confidence interval of the geometric mean ratio of the test to reference formulation for that pharmacokinetic parameter fell within the range of 80.00%–125.00%, the test and reference formulations were considered bioequivalent for that parameter. When the within-subject standard deviation of a pharmacokinetic parameter (C_max_, AUC_0-t_, or ${\text{AUC}}_{0-\infty }$ ) for the reference formulation was 0.294 or greater, the RSABE method recommended by the FDA was used for evaluation. If the point estimate of the geometric mean ratio of the test to reference formulation for that pharmacokinetic parameter was within the range of 80.00%–125.00%, and the critical bound value (The critical bound is the 95% upper confidence bound for ${\left({\bar{Y}}_{T}‐{\bar{Y}}_{R}\right)}^{2}$ - $\theta s_{\text{WR}}^{2}$ . ${\bar{Y}}_{T}$ and ${\bar{Y}}_{R}$ are the means of the In-transformed PK endpoint (AUC and/or C_max_) obtained from the BE study for the test and reference products, respectively, S_WR_ means Within-individual standard deviation of the Reference formulation. The scaled bioequivalence limit θ is adjusted for the reference formulation variability, where θ=(Ln(1.25)/ $\sigma_{\text{WO}}$ )2; $\sigma_{\text{WO}}$ is a regulatory constant defined by the FDA as 0.25.) (US Food and Drug Administration, 2021) was less than or equal to zero, the test and reference formulations were considered bioequivalent for that parameter. The difference in T_max_ between the two formulations was compared using the Wilcoxon signed-rank test.

## 3 Results

### 3.1 Volunteers characteristics

A total of 80 subjects were screened in this study. 48 failed the screening process, and 32 participants were randomly assigned to the trial groups, with mean (±SD) values for age, Height, weight, and BMI of 33.1 ± 9.09 years (range 19–50 years), 168.81 ± 6.19 cm (range 154–181 cm), 64.21 ± 6.77 kg (range 52.1–78.9 kg), and 22.49 ± 1.60 kg/m^2^ (range 19.2–25.7 kg/m^2^), respectively. Table 1 presents the demographic results of the two groups. During the trial, 30 subjects (93.8%) completed all 4 treatment Periods of pharmacokinetic blood sample collection as per the protocol. 2 subjects (6.2%; R006, R031) voluntarily withdrew from the trial due to personal reasons on the day before the fourth Period administration and on the day before the third Period administration respectively.

**TABLE 1:** Demographic information of the subjects (Mean ± SD).

Parameters	A group	B Group
	T-R-T-R (n = 16)	R-T-R-T (n = 16)
Age (years)	34.60 ± 8.19	31.60 ± 9.95
Height (cm)	169.38 ± 5.65	168.25 ± 6.83
Weight (kg)	63.93 ± 6.82	64.49 ± 6.94
BMI (kg/m^2^)	22.25 ± 1.66	22.74 ± 1.55

SD: Standard deviation, BMI: body mass index.

### 3.2 Method validation

The intra-batch and inter-batch accuracy and precision of the method were evaluated in at least three analytical batches over at least 2 days. The data are presented in Table 2. The results showed that the overall coefficient of variation (%CV) of the measured concentrations of quality control samples (LQC, MQC1, MQC2, and HQC) at each concentration level was ≤15.0% (≤20.0% for LLOQ QC), and the overall mean of the measured concentrations of quality control samples at each concentration level deviated from the theoretical concentration by ≤ ±15.0% (≤±20.0% for LLOQ QC). The results met the acceptance criteria.

**TABLE 2:** Intra-batch and inter-batch of accuracy and precision of onradivir.

QC concentration (ng/mL)	Intra-batch (N = 6)			Inter-batch (N = 18)		
	Mean ± SD (ng/mL	Precision (CV	Accuracy (%RE)	Mean ± SD (ng/mL)	Precision (CV%)	Accuracy (%RE)
4.00	4.24 ± 0.44	10.3	5.9	4.44 ± 0.42	9.4	10.9
12.0	12.1 ± 0.41	3.4	0.8	12.3 ± 0.57	4.6	2.8
300	311 ± 6.62	2.1	3.8	308 ± 7.98	2.6	2.7
5000	4,680 ± 106	2.3	−6.4	4,800 ± 171	3.6	−4.1
7500	7220 ± 115	1.6	−3.8	7200 ± 381	5.3	−3.4

SD: Standard deviation, CV: Coefficient of variation, RE: Relative error.

Six different sources of blank matrices were used to prepare LQC and HQC concentration samples. Each concentration level was repeated six times for matrix effect evaluation. The data are shown in Table 3. The results indicated that the %CV of the matrix factor (MF) of the analyte normalized by the internal standard calculated from each individual source of plasma matrix was ≤15%, and the %RE of accuracy was within ±15%, suggesting that different matrices did not affect the quantitative results of the analyte.

**TABLE 3:** Matrix effect investigation.

Blank matrix number	LQC (12.0 ng/mL)		HQC (7,500 ng/mL)	
	Mean ± SD	%RE	Mean ± SD	%RE
Matrix-1	12.5 ± 10.1	4.4	7277 ± 2.5	−3.0
Matrix-2	11.8 ± 0.8	−1.7	7210 ± 2.8	−3.9
Matrix-3	12.0 ± 3.4	0.3	7653 ± 1.1	2.0
Matrix-4	11.8 ± 0.8	−1.7	7503 ± 1.8	0.0
Matrix-5	11.8 ± 4.7	−1.9	7740 ± 3.5	3.2
Matrix-6	11.9 ± 2.1	−0.6	7730 ± 1.7	3.1

The extraction recovery rate of onradivir in plasma was calculated based on the peak area ratio. The results showed that the recovery rates at the concentrations of LQC, MQC and HQC were 107.6%, 89.8% and 99.9% respectively. The overall recovery rate of the internal standard was 99.9%. The %RE of each concentration in 6 parallel samples in the normal matrix was no more than 15.0%. There was no concentration-related trend in the average recovery rate of each concentration of the quality control samples. To ensure the stability of the detection process, this study evaluated the reliability of 10-fold dilution and the stability of the analyte in plasma, including 24-h room temperature storage, 5 freeze-thaw cycles at −20 °C and −80 °C, 37 days of storage in a −20 °C refrigerator, 228 days of storage in a −80 °C refrigerator, and the stability of the treated samples after storage at 4 °C for 118 h. All results met the acceptance criteria.

### 3.3 Biological sample testing

**FIGURE 2:**
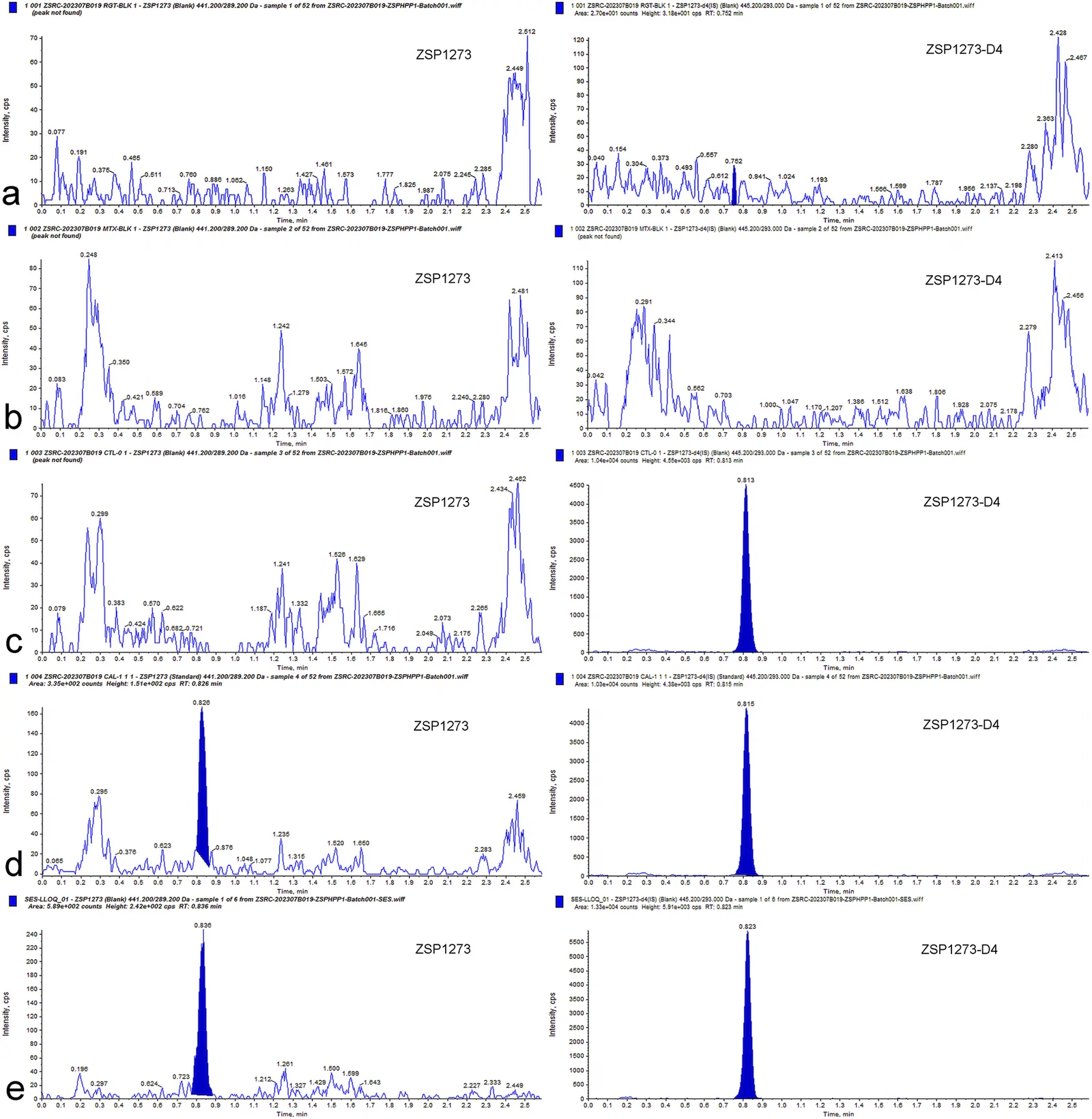
Representative chromatogram of ZSP1273 (onradivir) and IS in human blank plasma at **(a)** Blank reagent, **(b)** normal plasma, **(c)** Blank plasma with IS sample, **(d)** LLOQ sample, and **(e)** subject sample.

The LC-MS/MS method is a highly sensitive and highly specific detection method using isotopes as internal standards. The pretreatment of plasma samples adopts the protein precipitation method, which has a simple processing procedure and is particularly suitable for routine analysis of a large number of samples. The representative chromatogram of onradivir (number ZSP1273) and IS (number ZSP1273-D4) in human blank plasma is shown in Figure 2.

### 3.4 Pharmacokinetics

**FIGURE 3:**
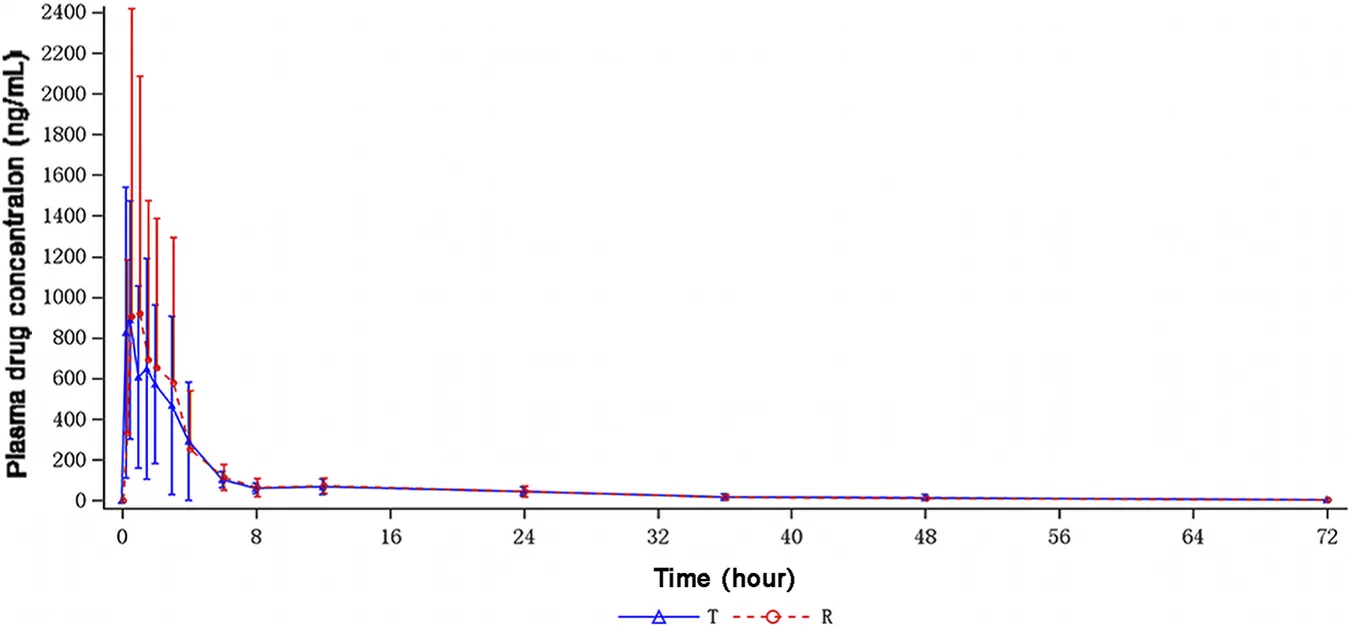
Mean plasma concentration-time profiles of onradivir after oral administration of 200 mg T/R to 32 healthy Chinese male subjects.

The mean plasma concentration-time profiles of onradivir following a single oral dose of 200 mg as granules and tablets are presented in Figure 3, with the corresponding main pharmacokinetic parameters summarized in Table 4. The results demonstrated comparable elimination half-lives (t1/2) between the two formulations, indicating that onradivir follows first-order elimination kinetics *in vivo*. That is, the drug is eliminated at a constant proportion relative to the instantaneous plasma concentration (proportional elimination), and this elimination characteristic is independent of the formulation type. However, a statistically significant difference in time to peak concentration (T_max_) was observed between the two formulations (P < 0.05), with the granule formulation showing a shorter median Tmax compared with the tablet.

**TABLE 4:** PK parameters of onradivir (Mean ± SD).

PKPS	Granule	Tablet
t_1/2_ (h)	13.96 ± 8.29	13.67 ± 8.32
T_max_ (h)	0.75 (0.25, 4.00)	2.00 (0.50, 6.00)
C_max_ (ng/mL)	1,374.30 ± 662.21	2028.70 ± 1,562.83
AUC_0-t_ (h·ng/mL)	4,465.96 ± 1,470.76	4,602.36 ± 1,693.73
${\text{AUC}}_{0-\infty }$ (h·ng/mL)	4,687.69 ± 1,666.04	4,737.15 ± 1,656.02
CL/F (mL/h)	47925.50 ± 16027.89	47642.63 ± 17611.51
${\text{MRT}}_{0-\infty }$ (h)	13.59 ± 8.05	12.42 ± 6.09

In the third period (R) of subject R002, the first period (R) of R010, R014, and R027, the second period (T) of R014, and the fourth period (T) of R020, AUC_0-t_ is less than 80% of ${\text{AUC}}_{0-\infty }$ . ${\text{AUC}}_{0-\infty }$ in this period is not included in PKPS. T_max_ is described as median (min, max).

### 3.5 Bioavailability assessment

A total of 32 healthy subjects completed the fasting study. The Within-individual standard deviation for C_max_ of the reference formulation were 0.667 (CV = 74.91%), respectively, indicating high variability for C_max_. Therefore, the RSABE method was used for the bioequivalence evaluation of C_max_. The within-subject standard deviations and coefficients of variation for AUC_0-t_ and ${\text{AUC}}_{0-\infty }$ were 0.180% and 18.11%, and 0.154% and 15.47%, respectively. These parameters exhibited lower variability, and thus the ABE method was used for their bioequivalence evaluation.

The point estimate of the least-squares geometric mean ratios for C_max_ 81.58%, its 95% upper confidence bound, at −0.1769, was <0, and the point estimate of the least-squares geometric mean ratio is 81.58%, fell within the 80.00%–125.00% range. The geometric mean ratios and 90% confidence intervals for AUC_0-t_, and ${\text{AUC}}_{0-\infty }$ of the test formulation relative to the reference formulation were 98.13% (93.91%, 102.54%), and 97.52% (93.22%, 102.03%), respectively (Table 5). The geometric mean ratios and 90% confidence intervals for AUC_0-t_ and ${\text{AUC}}_{0-\infty }$ also fell within the range of 80.00%–125.00%.

**TABLE 5:** Bioavailability parameters and evaluation methods.

Parameters	C_max_ (ng/mL) (N = 32)	AUC_0-t_ (h*ng/mL) (N = 32)	${\text{AUC}}_{0-\infty }$ (h*ng/mL) (N = 32)
T/R (%)	81.58	98.13	97.52
90% CI	70.41, 94.52	93.91, 102.54	93.22, 102.03
Methods for determining bioequivalence	RSABE	ABE	ABE

In the third period (R) of subject R002, the first period (R) of R010, R014, and R027, the second period (T) of R014, and the fourth period (T) of R020, AUC_0-t_ is less than 80% of ${\text{AUC}}_{0-\infty }$ . ${\text{AUC}}_{0-\infty }$ in this period is not included in BAS.

For the R002 subjects in the third period (R), the R010, R014 and R027 subjects in the first period (R), the R014 subjects in the second period (T), and the R020 subjects in the fourth period (T), the AUC__%Extrap_ was greater than 20% in all cases. Their ${\text{AUC}}_{0-\infty }$ was included in the sensitivity analysis. The results showed that the least squares geometric mean ratio of ${\text{AUC}}_{0-\infty }$ and its 90% confidence interval was 97.68% (93.14%, 102.44%), which fell within the range of 80.00%–125.00%, consistent with the main analysis results.

### 3.6 Taste evaluation

Palatability was assessed using a visual analogue scale (VAS) scoring system. The results showed that 75.8% of subjects had a palatability VAS score of 50 or higher for the drug, indicating good acceptability. The mean VAS score for the gritty sensation was 47.3, with a median of 50.0. During the trial, no subjects refused to take the medication, and there were no instances of subjects spitting out the drug during administration or vomiting after swallowing. Additionally, no subjects reported feeling nauseous during the medication intake process. These findings suggest that the test formulation, onradivir granules, has an acceptable taste that does not negatively impact medication intake.

### 3.7 Safety

No clinically significant abnormalities were observed in the physical examinations of all subjects, and vital signs remained stable throughout the study. A total of 32 subjects were included in the safety analysis set, with 62 administrations in the test (granule) group and 63 administrations in the reference (tablet) group. A total of 16 adverse events (AEs) occurred in 13 subjects (10.4%). In the T group, 2 subjects (3.2%) experienced 2 AEs, while in the R group, 11 subjects (17.5%) experienced 14 AEs. For treatment-related (AEs), 2 subjects (3.2%) experienced 2 AEs episodes in the T group, while 7 subjects (11.1%) experienced 8 AEs episodes in the R group. No statistically significant difference was observed between the two groups (P > 0.05). All adverse events were graded according to CTCAE version 5.0. Two subjects had 2 AEs (both with abnormal ST segment on ECG), which were graded as severity level 2 and resolved without treatment. The remaining AEs were graded as level 1. There were no AEs that led to participant withdrawal. The status of all AEs was either “resolved (2 AEs),ˮ “recovered (13 AEs),ˮ or “unknown due to loss to follow-up (1 AE).ˮ No subjects withdrew due to adverse events, and no serious adverse events or fatalities occurred.

## 4 Discussion

1. Evaluation of results for highly variable drugs. Early pharmaceutical studies indicated that onradivir tablets exhibit BCS Class IV characteristics (low solubility, low permeability), Oral absorption of such drugs is generally restricted by both dissolution and intestinal permeability, posing greater challenges to bioequivalence evaluation. Phase I clinical trial (Hu et al., 2021) results showed that C_max_ was achieved within 0.5–2.0 h after administration, with an average plasma elimination half-life (t1/2) ranging from 12.08 to 34.98 h. Notably, a high intrasubject coefficient of variation was observed for C_max_. For conventional bioequivalence studies, the ABE approach is typically employed. However, in accordance with the relevant regulatory requirements of the US Food and Drug Administration (2021), U.S. Department of Health and Human Services and Food and Drug Administration (2026), National Medical Products Administration of China (2018), when the individual coefficient of variation (CVw) of C_max_ and/or AUC reaches 30% or more, the drug is considered to be highly variable (S_WR_ ≥ 0.294), and the RSABE method can be adopted. A partial or complete replicated crossover design can be used, as it will adjust the equivalence limits based on the actual individual variations of the reference formulation, thereby reducing unnecessary subject exposure while maintaining the type I error rate at the nominal level (Dav et al., 2012). Accordingly, this trial adopted a four-period, two-sequence, replicate crossover design to minimize inter-subject variability. Following a single 200 mg oral dose of either the onradivir granules or onradivir tablets under fasting conditions, the within-individual standard deviation and coefficient of variation of the reference formulation’s C_max_, AUC_0-t_ and ${\text{AUC}}_{0-\infty }$ were 0.6674% and 74.91%, 0.1796 and 18.11%, 0.1538% and 15.47%. These results confirmed high variability in C_max_ for onradivir tablets. This is in agreement with earlier findings. For AUC_0-t_ and ${\text{AUC}}_{0-\infty }$ (S_WR_ <0.294), ABE was used. Both the GMRs and 90% confidence intervals for AUC parameters were within 80.00%–125.00%, confirming bioequivalence. Since the S_WR_ for C_max_ (≥0.294) met the regulatory threshold, RSABE was applied for C_max_ evaluation. S_WR_ of C_max_ was 0.667 exceeded the cutoff of 0.294. C_max_ met both acceptance criteria: its 95% upper confidence bound, at −0.1769, was <0, and the point estimate of the least-squares geometric mean ratio is 81.58%, fell within the 80.00%–125.00% range, meeting the RSABE equivalence determination criteria, demonstrating bioequivalence.
2. The pharmacokinetic parameters of the reference preparation in this study were compared with those of the previously marketed onradivir tablets (Hu et al., 2021). It was found that there was no statistically significant difference in the main parameter of bioavailability, C_max_, while AUC_0-t_ and ${\text{AUC}}_{0-\infty }$ showed statistically significant differences (P < 0.05). The results are presented in Table 6. Taking AUC_0-t_ as an example, the reference literature reported that after a single oral administration of onradivir tablets, within the dose range of 100–1200 mg, the mean plasma level of AUC_0-t_ and ${\text{AUC}}_{0-\infty }$ for ZSP1273 increased with the increase in dose. The AUC_0-t_ values for the 100, 200, and 400 mg dose ranges were 1879.33 ± 785.41, 6,347.47 ± 2,097.01, and 8,855.85 ± 1,686.51 h·ng/mL, respectively. In this study, the AUC_0-t_ for the 200 mg dose was 4,602.36 ± 1,693.73 h·ng/mL, which was more in line with the dose-linear relationship. The reason for the parameter differences might be due to the difference in the number of participants in the trials. The literature used 10 participants (including 2 placebo) to complete each dose group, with a relatively small number of participants. In contrast, this study used 30 participants to complete the parameter verification for the 200 mg dose, theoretically making the pharmacokinetic parameters closer to the true values.
3. Onradivir belongs to a new type of inhibitor targeting the PB2 subunit of the influenza virus RNA polymerase. It is the first approved PB2-targeted anti-influenza drug worldwide. Currently, apart from onradivir, the PB2 inhibitors that have entered the clinical stage include Pimodivir (VX-787) and CC-42344. Pimodivir, as the first PB2 inhibitor to enter clinical trials, has a C_max_ of approximately 1,212 ng/mL after a single oral dose of 600 mg, and an AUC_0–12h_ of approximately 3,709 ng·h/mL (Deleu et al., 2018). Onradivir, by modifying the structure of Pimodivir, has achieved a dual improvement in metabolic stability and antiviral activity (Hu et al., 2021). In the clinical trial of healthy subjects, onradivir showed a C_max_ of approximately 5463 ng/mL after a single 600 mg administration, and an AUC_0-t_ of approximately 17867 ng·h/mL (Hu et al., 2021). After dose normalization, its exposure level was significantly higher than that of Pimodivir. CC-42344 is another PB2 inhibitor in the II phase, but the specific C_max_, AUC, and other key PK parameters have not been fully disclosed in the public literature.
4. Considerations on pharmacokinetic studies in pediatric populations. Epidemiological data indicate that children under 5 years of age account for 50%–60% of influenza-related outpatient visits (World Health Organization, 2023), with the risk of severe disease inversely correlated with age (OR = 3.2, 95% CI: 2.1–4.8) (Centers for Disease Control and Prevention, 2023a). This significant disparity in disease burden underscores the urgent need, from a drug development perspective, to establish antiviral treatment strategies tailored for pediatric use. Pediatric dosing requires weight-based adjustments, imposing higher demands on drug formulations and ease of administration. Currently, the marketed onradivir tablet is unsuitable for children, prompting the development of a pediatric-friendly granule formulation based on the tablet. Typically, pediatric PK studies are preceded by adult PK studies to confirm that systemic drug exposure correlates with therapeutic outcomes. When the disease progression is similar between pediatric and adult populations, and the drug has been thoroughly studied and approved for adults, its use may be extrapolated to pediatric populations. Nevertheless, a more robust approach involves collecting PK data across a broad age range to assess age-related effects and guide dose adjustments (US Food and Drug Administration, 2022; Dunne et al., 2011). Specifically, we have expanded the discussion as follows: This study compares the bioavailability of the granule formulation with the approved tablet in healthy Chinese adult male subjects. The results provide initial formulation-bridging evidence to support the clinical development of the pediatric-friendly granule formulation. However, direct extrapolation of adult PK data to children is not straightforward due to substantial physiological differences between adults and children; therefore, independent pharmacokinetic and safety studies in the target pediatric population remain essential. Another critical factor in pediatric drug development is palatability, which significantly influences treatment success. Children are highly sensitive to taste, odor, and texture, and poor palatability may lead to refusal, vomiting, or treatment discontinuation, compromising efficacy. Studies show that approximately 30%–50% of children refuse medication due to unpleasant taste (European Medicines Agency, 2006), while 20%–40% of doses are wasted or spit out. Regulatory agencies such as the U.S. FDA mandate scientific palatability assessments in pediatric drug development to ensure acceptability and adherence (US Food and Drug Administration, 2016). In this trial, the investigational onradivir granule demonstrated moderate taste acceptability, posing no barriers to administration.
5. This study has the following limitations. First, only healthy male subjects were included. This was based on previous phase I data showing no sex-related PK differences for onradivir, and the primary objective of bridging the granule and tablet formulations in adults. However, since the granule form will be widely used in pediatric populations in the later stage, a clinical trial targeting pediatric patients with the target indication should include different genders to evaluate the safety and efficacy of the formulation in this population. Second, the study was conducted under fasting conditions; however, previous tablet data indicated that food had minimal effect on onradivir exposure, and a separate food-effect study of the granule formulation (Registration Number: CTR20243617) was completed by another team in 2024; Thirdly, the taste assessment in this study used a self-made VAS questionnaire, whose design referred to the facial expression scale method and the evaluation methodology literature on the gritty sensation of granules (Schluterman et al., 2024; Lopez et al., 2016). However, this tool was used for exploratory purposes and did not undergo complete reliability and validity tests.

**TABLE 6:** Comparison of the main pharmacokinetic parameters of onradivir tablets.

PKPS	Tablet	Tablet (Hu et al., 2021)
T_max_ (h)	2.00 (0.50, 6.00)	1.50 (3, 0.75)
C_max_ (ng/mL)	2028.70 ± 1,562.83	2393.63 ± 993.90
AUC_0-t_ (h·ng/mL)	4,602.36 ± 1,693.73	6,347.47 ± 2097.01
${\text{AUC}}_{0-\infty }$ (h·ng/mL)	4,737.15 ± 1,656.02	6,631.07 ± 2057.83
t_1/2_ (h)	13.67 ± 8.32	20.14 ± 7.63
CL/F (mL/h)	47642.63 ± 17611.51	33341.85 ± 12065.41
${\text{MRT}}_{0-\infty }$ (h)	12.42 ± 6.09	15.57 ± 5.78

## 5 Conclusion

In this study, the onradivir granules demonstrated favorable safety profiles following single-dose oral administration under fasting conditions in healthy subjects. The formulation exhibited excellent palatability with acceptable grittiness, making it suitable for pediatric use. Bioequivalence was established between the onradivir granules and the marketed onradivir tablets. Additionally, since influenza caused by the influenza A virus exhibits similar disease characteristics, pharmacological effects of the drug, and expected therapeutic responses in both pediatric and adult populations, pharmacokinetic data obtained from adults, following good clinical research design principles and regulatory requirements, can be extrapolated using strategies, primary quantitative methods, and application scenarios to provide reference for drug development in different pediatric age groups. This approach can help reduce the number of pediatric subjects and research steps, thereby optimizing the development process of pediatric formulations.

## Data Availability

The data that support the findings of this study are not openly available due to privacy/ethical restrictions. All of Study Protocol, Informed Consent Form, statistical analysis plan, and Clinical Study Report can also be available upon request. The information and data presented in the article can be obtained by contacting the corresponding author.
