# Supplementary material for: Bioavailability of a novel anti-influenza agent, onradivir granules, in healthy Chinese adults: a first-in-human study
Source: Front Pharmacol. 2026 Sep 1;17:1905152. doi: 10.3389/fphar.2026.1905152 (PMC13575753; doi:10.3389/fphar.2026.1905152)
Supplement: Supplementary file 1 [file DataSheet1.pdf]

Supplementary Material 1

VAS scoring scale for onradivir granule

Subject screening No.:

Subject randomization No.:

Completion date: Period: □ Period 1 □ Period 2 □ Period 3 □ Period 4

VVAS Score for Drug Palatability (Tick only one option matching your taste experience of drug suspension)

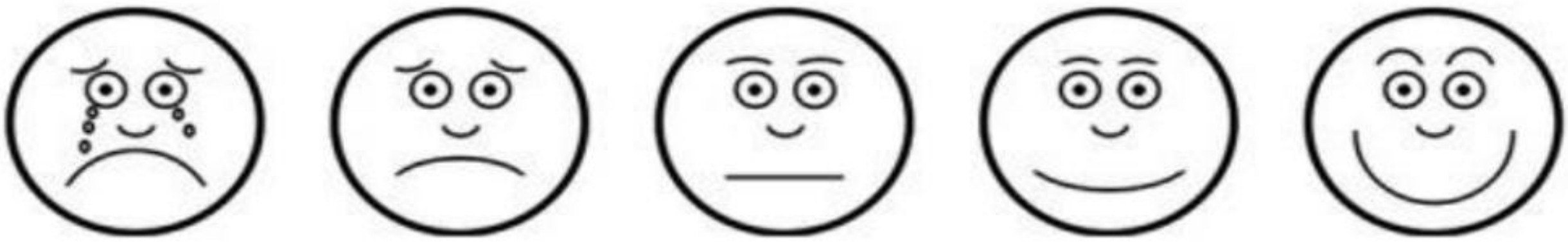

□ Score 0<br>very bad	□ Score 20<br>bad	□ Score 50<br>not nice-not bad	□ Score 80<br>nice	□ Score 100<br>very nice

VAS Score for Gritty Sensation of Drug：Gritty sensation score: ____________ points

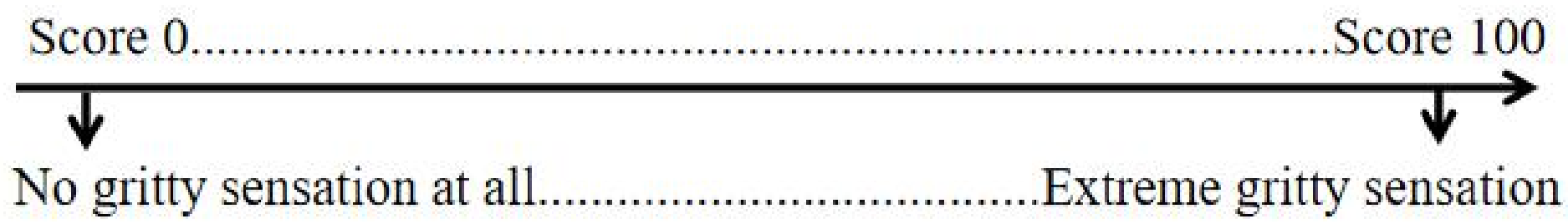

Adverse Reactions & Abnormal Conditions During Dosing(Tick Yes / No for each item separately)：

a. Refused to take medication □ No □ Yes

b. Spat out the drug □ No □ Yes

c. Vomiting after swallowing □ No □ Yes

d. Nausea □ No □ Yes

e. Other conditions □ No □ Yes, please describe:

Important Notes：

1. This scale is used to record subjects' status after taking granular preparation, before mouth rinsing in each treatment cycle.
2. Drug palatability evaluation includes texture, volume, taste, aftertaste and odor of drug suspension. Combined with facial expression scale, a 5-grade classified VAS scale is applied.
3. Gritty sensation evaluation focuses on particle size and particle quantity in suspension, adopting a 0–100 point VAS scale.
